# Statistical learning in visual search reflects distractor rarity, not only attentional suppression

**DOI:** 10.3758/s13423-022-02097-x

**Published:** 2022-04-20

**Authors:** Dirk Kerzel, Chiara Balbiani, Sarah Rosa, Stanislas Huynh Cong

**Affiliations:** Faculté de Psychologie et des Sciences de l’Education, Université de Genève, 40 Boulevard du Pont d’Arve, 1205 Genève, Switzerland

**Keywords:** Visual search, Attentional selection, Attentional suppression, Statistical learning

## Abstract

In visual search tasks, salient distractors may capture attention involuntarily, but interference can be reduced when the salient distractor appears more frequently on one out of several possible positions. The reduction was attributed to attentional suppression of the high-probability position. However, all previous studies on this topic compared performance on the high-probability position to the remaining positions, which had a low probability of containing the distractor. Therefore, it is not clear whether the difference resulted from reduced interference on the high-probability position or from increased interference on the low-probability positions. To decide between these alternatives, we compared high-probability and low-probability with equal-probability positions. Consistent with attentional suppression, interference was reduced on the high-probability position compared with equal-probability positions. However, there was also an increase in interference on low-probability positions compared with equal-probability positions. The increase is in line with previous reports of boosted interference when distractors are rare. Our results show that the experimental design used in previous research is insufficient to separate effects of attentional suppression and those of distractor rarity.

**Supplementary Information:**

The online version contains supplementary material available at 10.3758/s13423-022-02097-x.

## Introduction

Humans are confronted with more visual information than they can possibly deal with. Therefore, it is thought that attention focusses processing resources on relevant information through a variety of mechanisms, such as noise reduction or signal enhancement (Carrasco, 2011). To guide attention to relevant information, stored target characteristics enhance corresponding visual features (Bundesen, 1990; Desimone & Duncan, 1995; Eimer, 2014; Huynh Cong & Kerzel, 2021; Kruger et al., 2017; Liesefeld et al., 2018; Luck et al., 1997; Schneider, 2013; Wolfe, 2021). Although top-down control of attention seeks to focus on relevant information, irrelevant distractors may interfere with visual search because of their bottom-up saliency. The interaction of top-down and bottom-up guidance of attention has been investigated in the additional singleton paradigm developed by Theeuwes (1991, 1992, 2010). In a typical variant of the additional singleton paradigm, participants searched for a unique shape among uniform nontarget shapes. On some trials, one of the nontarget shapes was a color different from the others. For instance, participants searched for a green circle target among green diamond nontargets and on some trials, the color of one of the diamonds was changed from green to red. Search times increased on distractor-present compared with distractor-absent trials although the distractor could be ignored. It was suggested that the reason for the interference is bottom-up attentional capture by the salient distractor (for discussion of this idea, see Luck et al., 2021).

In addition, it was argued that attentional capture is shaped by trial history. Among the numerous effects of trial history reported in the literature (for review, see Awh et al., 2012; Lamy & Kristjánsson, 2013), effects of positional probabilities have received much interest lately (e.g., Ferrante et al., 2018; Goschy et al., 2014; Reder et al., 2003; Wang & Theeuwes, 2018b). Consistent with the pioneering work of Reder et al. (2003), two effects have been repeatedly reported when distractors appeared with a higher probability on one out of several positions (or regions). First, interference from the distractor on the high-probability position decreased compared with distractors presented on one of the remaining low-probability positions. Second, on distractor-absent trials, reaction times (RTs) increased when the target was shown on the high-probability distractor position compared with when it was shown on a low-probability distractor position. The reduced distractor interference on the high-frequency distractor position was attributed to the shielding of visual search from likely distractor positions (e.g., Goschy et al., 2014; Sauter et al., 2018) or altered distractor filtering (e.g., Ferrante et al., 2018), but by far the most frequent interpretation was that it resulted from attentional suppression (Di Caro & Della Libera, 2021; Di Caro et al., 2019; Kerzel & Huynh Cong, 2021; Liesefeld & Müller, 2021; Sauter et al., 2021; Sauter et al., 2019; van Moorselaar et al., 2020; van Moorselaar & Slagter, 2019; van Moorselaar & Theeuwes, 2021a, 2021b; Wang & Theeuwes, 2018a, 2018b, 2018c). Attentional suppression of the high-frequency distractor position was assumed to be learned through the repeated presentation of the distractor on the same position and served to reduce distractor interference. In most studies, the need to reduce distractor interference was large because experimental conditions were selected to maximize interference. For instance, the target features were unpredictable (e.g., Allenmark et al., 2019) or the distractors were from the same dimension as the target (e.g., Liesefeld et al., 2019), and in all cases the distractor was highly salient (e.g., Failing & Theeuwes, 2020). While attentional suppression of the high-probability distractor position decreased interference from distractors, it also impaired processing of targets on this position. On trials without distractor, targets on the high-probability distractor position were processed more slowly than targets on low-probability positions, suggesting that attentional suppression occurred.

In the present investigation, we asked whether the difference between high-probability and low-probability distractor positions can be unambiguously attributed to attentional suppression. The reason for the ambiguity is that distractor positions in previous research were either high probability or low probability, but a control condition with equal probability was missing. While the reduced distractor interference on the high-probability position was mostly attributed to attentional suppression, interference was only reduced with respect to the low-probability distractor positions, not a condition with equal-probability distractor placement. Therefore, it is possible that the difference was accounted for by increased interference on low-probability positions, not by reduced interference at the high-probability position. Consistent with this argument, previous research has demonstrated that interference increased when distractors appeared only on a small number of trials (Geyer et al., 2008; Müller et al., 2009; see also Ernst et al., 2020). However, none of the previous studies compared interference between rare and frequent distractor positions. It is therefore necessary to confirm that distractor rarity not only affects expectations about how often a distractor appears, but also where it appears.

## Experiments 1 and 2

**Fig. 1:**
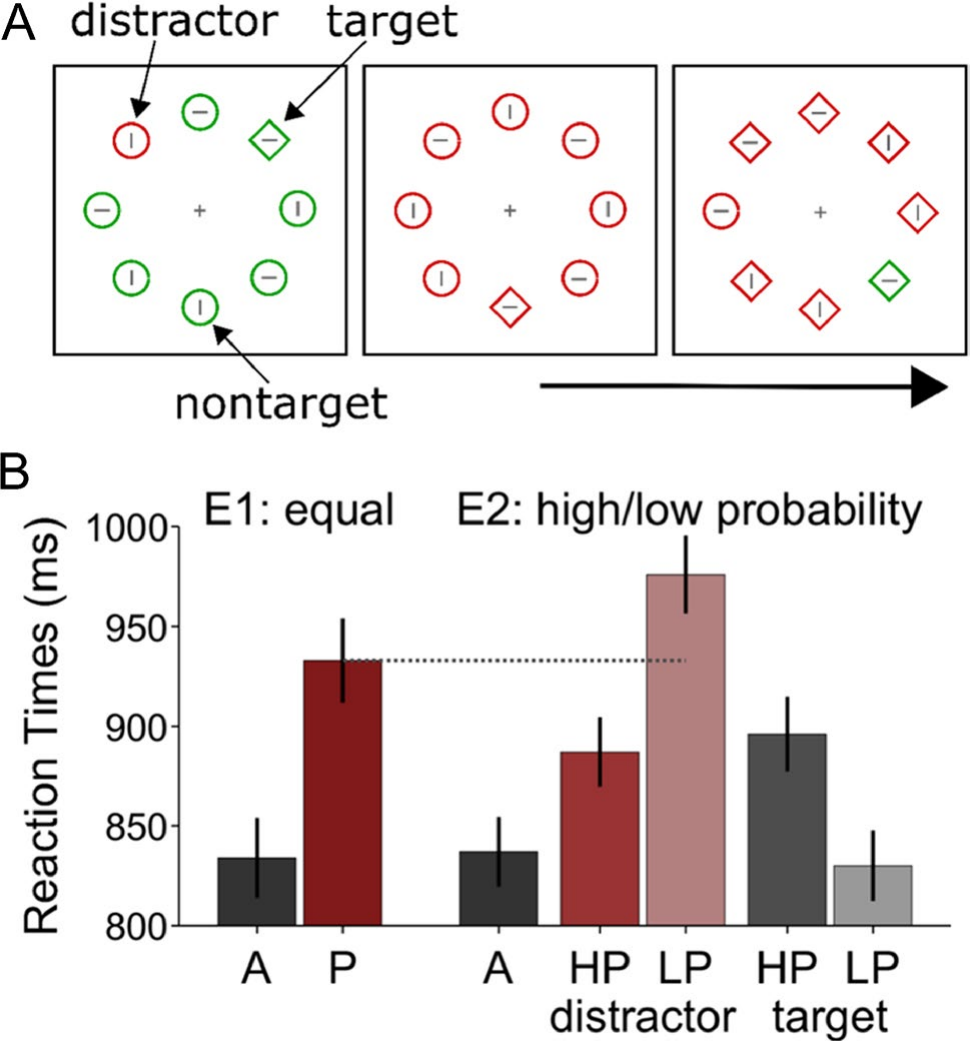
Panel **a** shows an illustration of the experimental stimuli (drawn to scale). Panel **b** shows the reaction time results from Experiments 1 and 2 (E1, E2). In Experiment 1, the probability of distractor or target presentations on each of the eight positions was equal. In Experiment 2, the distractor or target could be shown on the high-probability distractor position or on one of the low-probability distractor positions. Black or gray bars indicate means from distractor-absent conditions. Reddish bars indicate means from distractor-present conditions. Error bars show the between-participant standard error. A = distractor-absent; P = distractor-present; HP = high-probability distractor position; LP = low-probability distractor position. (Color figure online)

Therefore, we assessed contributions of attentional suppression and distractor rarity to the difference between high- and low-probability distractor positions. To this end, we compared interference with equal-probability distractor placement to interference with high- and low-probability distractor positions. We took a conservative approach and chose a between-participant design to avoid effects of order. However, it may be possible to run the comparison in a within-subject design because distractor learning was found to be short lived (Di Caro & Della Libera, 2021; Ferrante et al., 2018). The experimental stimuli (see Fig. 1a) and the design of the experiment with high- and low-probability distractor placement (see Table 1) were closely modeled on Wang and Theeuwes (2018b).

**Table 1:** Number of target and distractor presentations by position in Experiments 1 and 2 (E1, E2)

	E1: equal		E2: high/low	
position	target	distractor	target	distractor
1	~90	60	~53.1	312
2	~90	60	~95.3	24
3	~90	60	~95.3	24
4	~90	60	~95.3	24
5	~90	60	~95.3	24
6	~90	60	~95.3	24
7	~90	60	~95.3	24
8	~90	60	~95.3	24
sum	720	480	720	480

There were eight positions in the search display, which were labelled 1 to 8. For illustration, the high-probability distractor position in Experiment 2 was assigned to position 1, but the actual high-probability position was counterbalanced across participants. We experimentally controlled the distractor position, whereas the target position varied randomly. Thus, the actual number of target presentations on each position varied from participant to participant (indicated by the “~” symbol), whereas the actual number of distractor presentations did not. Note that target and distractor presentations on the same position occurred on different trials because the target shape was never in the distractor color.

If attentional suppression was the only cause for the difference between high- and low-probability distractor positions, then interference with equal-probability distractor placement should be similar to interference with distractors on the low-probability distractor position. This pattern of results would suggest that interference on the low-probability distractor positions corresponded to baseline interference, whereas interference with distractors on the high-probability positions was reduced because of attentional suppression. While this reasoning is implicit in all of the previous research, it is also possible that rarity boosted interference when distractors were presented on the low-probability positions. If effects of distractor rarity caused the difference between high- and low-probability distractor positions, interference with equal-probability distractor placement should be similar to interference with distractors on the high-probability distractor position. This pattern of results would suggest that there is no contribution of attentional suppression, but rarity would increase interference by distractors on the low-probability positions. This outcome is unlikely because results from distractor-absent trials show that target processing was impaired on the high-probability distractor position, which provides strong evidence for a contribution of attentional suppression. More likely, both attentional suppression and distractor rarity contribute to the difference between high- and low-probability distractor positions. In this case, interference with equal-probability placement would be intermediate.

### Methods

#### Participants

First-year psychology students at the University of Geneva participated for class credit. For the paired *t* test between the high- and low-probability condition, Wang and Theeuwes (2018b) found *t*(24) = 9.82, which translates into Cohen’s *d*_*z*_ = 2.00. Only four participants are necessary to find an effect of this size with an alpha of .05 and a power of 0.8 according to G*Power 3.1 (Faul et al., 2009). However, we were interested in between-participant comparisons. Müller et al. (2009, p. 5) compared groups of participants with frequent and infrequent distractor presentation. For this independent-samples *t* test, they reported *t*(22) = 3.23, which translates into Cohen’s *d*_*s*_ = 1.32. We settled on 40 participants per experiment, which would allow for the detection of differences with a minimal *t*(78) = 1.99 (two-tailed) and Cohen’s *d*_*s*_ = 0.63. The total sample size of 80 is close to the recommended 90 participants for expected effect sizes of 0.6 (Brysbaert, 2019). However, the data of one participant were removed because the choice error rate of 24% was much higher than in the rest of the sample (*M* = 6.2%, *SD* = 3.6%). The final sample had 40 participants in Experiment 1 (20 men, age: *M* = 20 years, *SD* = 1.4) and 39 in Experiment 2 (22 men, age: *M* = 20 years, *SD* = 1.5). All students reported normal or corrected-to-normal vision. The study was approved by the ethics committee of the Faculty of Psychology and Educational Sciences and was carried out in accordance with the Code of Ethics of the World Medical Association (Declaration of Helsinki). Informed consent was given before the experiment started.

#### Apparatus

Stimuli were presented at 60 Hz on one of four LCD monitors (Philips 242G, Philips, Amsterdam, The Netherlands). The CIE 1931 xyY coordinates of the stimuli on one of those monitors were red = (0.655, 0.321, 52.4), green = (0.306, 0.602, 52.5), and gray = (0.316, 0.304, 51.7) with *Y* in cd/m^2^. The color coordinates on the other monitors were similar. A ColorCAL MKII colorimeter (Cambridge Research Systems, Rochester, Kent, UK) was used to measure the color coordinates. Viewing distance was approximately 66 cm. No chin rest or head restraint was used. The arrow keys of a regular keyboard served to collect the responses.

#### Stimuli

The background was black. A gray fixation cross (0.5° × 0.5°) was shown in the center of the screen. The eight geometric shapes in the search display were equally spaced on an imaginary circle at an eccentricity of 4° from the fixation cross (center to center). One shape was shown directly above, below, left, and right of central fixation. The circle and diamond had diameters of 1.5° and 1.7°, respectively, and were drawn in a linewidth of 0.07°. The gray lines inside the geometrical shapes were 1.2° long. The target had a unique shape, and the distractor had a unique color. The majority color and shape switched randomly. That is, the target was either the only circle among diamonds or the only diamond among circles. The majority color was either red or green and the distractor, if present, was the other color. The target was always in the majority color so that target and distractor never coincided (see Fig. 1a).

#### Procedure

A fixation period of 0.75–1.0 s elapsed before the search display was shown. The search display and the fixation cross remained visible until a key press was detected. Participants were instructed to maintain fixation and to indicate the orientation of the line inside the target shape by pressing the left-arrow key for horizontal and the up-arrow key for vertical. Participants were told to ignore shapes in a unique color because the target would never be in a unique color. Participants were asked to respond as rapidly as possible while keeping the error rate below 10%. Feedback about the median RTs and the percentage of errors was given after blocks of 60 trials. The feedback was shown for at least 2,000 ms, and participants were free to continue the experiment at their own pace by pressing a key. Choice errors and RTs outside the response window of 2,000 ms were signaled to the participant by immediate visual feedback. The experiment started with a training block of at least 60 trials. Participants could continue to run practice trials until they felt comfortable with the task (*M* = 80 trials, *SD* = 42). Sixty-one participants completed between 60 and 70 trials, and 19 participants between 111 and 240 trials. At the end of the experiment, we asked all participants to indicate a high-frequency distractor position. Participants were shown an illustration indicating the eight stimulus locations and marked the location where they thought the distractor had been presented more frequently. Participants were run in groups of up to four students in the same lab. To avoid visual interference, participants were separated by screens.

#### Design

The target shape and color varied randomly from trial to trial. Target and distractor never coincided. There were 720 trials in each experiment. As shown in Table 1, a distractor was presented on 480 trials, or 67% of all trials. In Experiment 1, the probabilities of target and distractor presentation corresponded to random placement. That is, the target was presented on ~12.5% of the trials on each of the eight positions (i.e., 90 trials), and the distractor was presented on 8.3% of trials on each of the eight positions (i.e., 60 trials). In Experiment 2, one position had a higher probability of containing the distractor. The distractor was presented on 43.3% of all trials (i.e., 312 trials) on the high-probability distractor position compared with 3.3% (i.e., 24 trials) on one of the low-probability distractor positions. If only distractor-present trials are considered, the high-probability position occurred on 65% of trials compared with 45% for the seven low-frequency positions. Because target and distractor never coincided, the probability of a target being presented on the high-probability distractor position was reduced (~7.4% or 53.1 trials) compared with low-probability distractor positions (~13.2% or 95.3 trials). However, Failing et al. (2019) demonstrated that the pattern of statistical learning did not differ if the probability of target appearance on the high-probability distractor location was equal to the remaining positions. The experimental program ensured the number of distractor presentations per position shown in Table 1, whereas the target position was selected randomly. Thus, the number of target presentations per position varied from participant to participant, which is indicated by the tilde symbol (~). The experiment was run in three blocks of 240 trials.

### Results

#### Reaction times

Before calculating individual mean RTs, we removed choice errors (Exp. 1: 5.9%, Exp. 2: 6.2%), responses outside the response window of 2,000 ms (Exp. 1: 2.0%, Exp. 2: 1.3%) and trials with RTs that were 2.5 standard deviations above the respective condition mean (between 2% and 3%). Mean RTs are shown in Figure 1B. To rule out that the general search performance differed between Experiments 1 and 2, we compared distractor-absent trials. By independent-samples *t* test, RTs in the distractor-absent conditions of Experiments 1 and 2 did not differ (834 vs. 837 ms), *t*(77) = 0.12, *p* = .91, Cohen’s *d*_*s*_ = 0.03.

Next, we evaluated interference from the distractor separately for each experiment. Interference refers to the difference between distractor-present and distractor-absent trials. With equal-probability distractor placement in Experiment 1, a paired *t* test showed that RTs were 99-ms longer on distractor-present than on distractor-absent trials (933 vs. 834 ms), *t*(39) = 20.22, *p* < .01, Cohen’s *d*_*z*_ = 3.20. With high- and low-probability distractor placement in Experiment 2, interference was 50 ms with distractors on the high-probability position (887 vs. 837 ms), *t*(38) = 9.54, *p* < .01, Cohen’s *d*_*z*_ = 1.53, and 139 ms with distractors on the low-probability position (976 vs. 837 ms), *t*(38) = 17.86, *p* < .01, Cohen’s *d*_*z*_ = 2.86. Consistent with previous research, we confirmed that interference was reduced with distractors on high- compared with low-probability positions (50 vs. 139 ms), *t*(38) = 15.62, *p* < .01, Cohen’s *d*_*z*_ = 2.5.

Our main interest was to determine whether the difference between high- and low-probability distractor positions resulted from reduced interference on the high-probability position or from increased interference on the low-probability positions. To assess these hypotheses, we compared interference with equal-probability distractor placement to interference with distractors on high- and low-probability positions. By independent-samples *t* tests, interference with equal-probability distractor placement was larger than with distractors on the high-probability position (99 vs. 50 ms), *t*(77) = 6.93, *p* < .01, Cohen’s *d*_*s*_ = 1.56, suggesting that the high-probability distractor position was suppressed. At the same time, interference with equal-probability distractor placement was smaller than with distractors on the low-probability position (99 vs. 139 ms), *t*(77) = 4.32, *p* < .01, Cohen’s *d*_*s*_ = 0.97, suggesting that interference at low-probability positions was boosted by distractor rarity.

Further, we ran additional analyses to show that we replicate effects of statistical learning from previous studies in Experiment 2. On distractor-absent trials, RTs were 66-ms longer with targets on the high-probability distractor position compared with targets on the low-probability distractor position (896 vs. 830 ms), *t*(38) = 6.33, *p* < .01, Cohen’s *d*_*z*_ = 1.01. This difference shows that the high-frequency distractor position was suppressed, which increased RTs when the target was shown on this location. On distractor-present trials, RTs increased with increasing distance from the high-probability distractor location (887, 943, 995, 992, 993 ms for distances 0–4, respectively), *F*(4, 152) = 35.71, *p* < .01, η_p_^2^ = .484, confirming a spatial gradient of distractor suppression. The effect of distance remained significant after exclusion of the high-frequency distractor position, *F*(3, 114) = 8.52, *p* < .01, η_p_^2^ = .183.

#### Choice errors

The analysis of percentages of choice errors confirmed the results from the analysis of RTs and showed no signs of speed–accuracy trade-off (see Supplemental Material A).

#### Assessment of awareness

The analysis of judgments of the most frequent distractor position in Experiment 2 confirmed results from previous work (see Supplemental Material B).

## Discussion

We replicated reduced interference on high- compared with low-probability distractor positions in Experiment 2. The previous literature mostly attributed the reduction to attentional suppression of the high-probability distractor position. However, it is possible that the difference between high- and low-probability distractor positions resulted from increased interference on the low-probability distractor positions, not from decreased interference on the high-probability position. To solve the ambiguity, we compared interference with high- and low-probability distractor positions in Experiment 2 to interference with equal-probability distractor placement in Experiment 1. If the difference between high- and low-probability distractor positions reflected only attentional suppression, interference with equal-probability distractor placement should be similar to interference with low-probability distractor positions. However, we found larger interference with low-probability distractor positions compared with equal-probability distractor placement, suggesting that distractor rarity boosted interference on low-probability distractor positions. Thus, previous conclusions regarding statistical learning and attentional suppression are at least partially incorrect because contributions of distractor rarity were not considered. Further, our results suggest that attentional suppression and distractor rarity contributed about equally. Relative to equal-probability distractor placement, interference decreased by 49% with distractors on the high-probability position (99 vs. 50 ms) and increased by 40% with distractors on the low-probability position (99 vs. 139 ms).

The present study suggests that attentional suppression only partially explains the difference between high- and low-probability distractor positions. In a similar vein, research using neural measures may have overestimated effects of attentional suppression. For instance, Wang et al. (2019) suggested that suppression of the high-probability distractor position occurred even before stimulus onset. Evidence for proactive attentional suppression was that contralateral alpha oscillations increased. In general, attentional suppression is associated with increased contralateral alpha oscillation, whereas the deployment of attention is associated with decreased contralateral alpha oscillations. However, the interpretation of lateralized alpha oscillations is plagued by ambiguities (Foster & Awh, 2019). As only the difference between hemispheres is evaluated, the results are consistent with two conflicting interpretations. Notably, it is possible to consider the reduced alpha oscillations in one hemisphere as increased alpha oscillations in the other hemisphere. Wang et al. (2019) suggested that the increased alpha oscillations resulted from attentional suppression of the high-probability distractor position in the contralateral visual hemifield. However, it may also be possible that participants deployed attention to the opposite side where only low-probability distractor positions were shown. In both cases, alpha oscillations would increase in the hemisphere contralateral to the high-probability position. Further doubts on the role of proactive attentional suppression come from a failure to replicate the prestimulus modulations of alpha oscillations (van Moorselaar et al., 2020). In addition, Wang et al. (2019) recorded event-related potentials to the distractor and observed a contralateral positivity at posterior electrodes PO7/PO8, which is referred to as P_D_. The P_D_ has been associated with distractor suppression (Burra & Kerzel, 2013; Hickey et al., 2009; Sawaki & Luck, 2010), but it suffers from a similar ambiguity as the lateralized alpha oscillations. A positivity contralateral to the distractor could also be a negativity contralateral to the stimuli on the other side of fixation (Kerzel & Burra, 2020). Similar to the present investigation, the neural measures mentioned here would tremendously benefit from a neutral condition which clarifies whether there was attentional suppression of one stimulus or enhancement of the stimulus in the other hemifield.

### Underlying mechanisms

Selective attention is responsible for the allocation of limited processing resources to possible target stimuli (Carrasco, 2011; Eimer, 2014; Gaspelin & Luck, 2018). That is, stimuli selected by attention receive more resources than others, which enhances processing of these stimuli. Conversely, attentional suppression is thought to prevent the allocation of attentional resources to distractor stimuli. As a result, the processing of salient distractor stimuli, as evaluated by a secondary probe task, may be worse than the processing of other nontarget stimuli (Gaspelin et al., 2015; but see Lien et al., 2021). In the context of the present study, attentional suppression of the high-frequency distractor position may have prevented the erroneous allocation of attentional resources to distractors appearing at this location. As a result, interference by the distractor decreased relative to equal distractor placement.

One may wonder whether attentional suppression of the high-probability distractor position increased the available processing resources at the remaining positions. Unfortunately, previous research does not provide a clear answer to this question. On the one hand, it was observed that increased neural suppression on distractor-present trials was associated with shorter RTs (Gaspar & McDonald, 2014). On the other hand, there was no indication of neural suppression when interference could be completely avoided (Barras & Kerzel, 2016). In the current study, a potential increase of available processing resources for unsuppressed locations makes a strong prediction for distractor-absent trials. If the available processing resources increase on low-probability distractor positions, search times should decrease for targets on these positions compared with equal-probability positions. However, this was not the case (830 vs. 834 ms), *t*(77) = 0.5, *p* = .86, Cohen’s *d*_*s*_ = 0.21. Therefore, it appears unlikely that the increased interference for distractors on low-probability positions resulted from an increase in processing resources.

In previous studies, the increased interference with rare compared with frequent distractor presentation was attributed to the reduced top-down incentive to suppress distractors when they were rare. However, these studies compared blocks of trials where distractors were rare with blocks of trials where distractors were frequent (Geyer et al., 2008; Müller et al., 2009). In the current research, the frequency of distractor-present trials was always the same and only the probability of the distractor position changed. Nonetheless, a similar mechanism may be at work. The top-down incentive to suppress distractors on low-probability positions was weak and therefore, less suppression was applied and interference by the distractor was stronger.

### The neutral condition in the cueing literature

The ambiguities discussed in the present article are reminiscent of a previous debate in the cueing literature. Peripheral cues result in better performance at the cued than at uncued locations (Posner & Cohen, 1984). However, the question was whether the difference reflects benefits at the cued location or costs at the uncued location (de Gonzaga Gawryszewski et al., 1987; Jonides & Mack, 1984). To decide between these alternatives, a neutral condition is needed, but there are several options for creating a neutral condition. For instance, a condition without cues could serve as neutral condition, but it may be that general alertness is lower without cues (Fan et al., 2002; Prasad et al., 2021). Another option is to present cues at all different locations, but many homogeneous cues facilitate target detection compared with a single cue (Schönhammer et al., 2020). Nonetheless, the general conclusion in the cueing literature was that the neutral condition is intermediate between performance at cued and uncued locations, suggesting that benefits and costs exist (Carrasco, 2011). In the present investigation, we arrive at a similar conclusion because the condition with equal-probability distractor placement yielded results that were intermediate between the low- and high-probability distractor positions. In contrast to the discussion in the cueing literature, however, there are far less doubts about characteristics of the baseline or neutral condition. Equal-probability distractor placement represents an almost natural reference point for high- and low-probability distractor placement.

### Conclusion

The recent literature on statistical learning has claimed that the reduction of distractor interference at high- compared with low-probability distractor positions reflects attentional suppression of the high-probability distractor position. While impaired target processing at this position on distractor-absent trials supports this claim, it cannot be ruled out that the rarity of distractors on low-probability positions also contributes. To test this idea, we compared interference on high- and low-probability distractor positions to equal-probability distractor placement. We found interference on low-probability distractor position to be larger than with equal-probability distractor placement, which confirms that distractor rarity boosted interference. Thus, the difference between high- and low-probability distractor positions reflects both attentional suppression and effects of distractor rarity. As contributions of distractor rarity were previously neglected, conclusions drawn in previous studies need to be revised.

## Supplementary Information

ESM 1 (DOCX 19 kb)
