# Supplementary material for: Statistical learning in visual search reflects distractor rarity, not only attentional suppression
Source: Psychon Bull Rev. 2022 Apr 20;29(5):1890–7. doi: 10.3758/s13423-022-02097-x (PMC9568448; doi:10.3758/s13423-022-02097-x)
Supplement: Supplementary file 1 — (DOCX 19 kb) [file 13423_2022_2097_MOESM1_ESM.docx]

**Supplemental Material A**

The analysis of percentages of choice errors confirmed the results from the analysis of RTs and showed no signs of speed-accuracy trade-off. The error percentages on distractor-absent trials did not differ between Experiments 1 and 2 (3.6% vs. 4.5%), *t*(77) = 1.50, *p* = .14, Cohen’s *d_s_* = 0.34, confirming that overall performance was similar.

Next, we evaluated differences in error percentages between distractor-present and -absent trials separately for each experiment. In Experiment 1, a paired t-test showed that 3.4% more errors occurred on distractor-present than -absent trials (7.0% vs. 3.6%), *t*(39) = 10.40, *p* < .01, Cohen’s *d_z_* = 1.64. Similarly, in Experiment 2, more errors occurred on distractor-present than -absent trials with distractors on the high-probability position (5.9% vs. 4.5%), *t*(38) = 3.74, *p* < .01, Cohen’s *d_z_* = 0.60, and also with distractors on the low-probability location (9.1% vs. 4.5%), *t*(38) = 7.69, *p* < .01, Cohen’s *d_z_* = 1.23. Interference was reduced with distractors on the high-probability compared to the low-probability positions (1.4% vs. 4.6%), *t*(38) = 6.08, *p* < .01, Cohen’s *d_z_* = 0.97.

Concerning the comparisons between Experiments 1 and 2, independent-samples t-tests showed that interference with equal-probability distractor placement was larger than with distractors on the high-probability position (3.4% vs. 1.4%), *t*(77) = 3.79, *p* < .01, Cohen’s *d_s_* = 0.85, and tended to be smaller than with distractors on the low-probability position (3.4% vs. 4.6%), *t*(77) = 1.83, *p* = .07, Cohen’s *d_s_* = 0.35.

Concerning suppression of target-processing in Experiment 2, we found that on distractor-absent trials, error percentages tended to be larger with targets on the high-probability distractor position compared with targets on the low-probability distractor position (5.7% vs. 4.3%), *t*(38) = 2.01, *p* = .052, Cohen’s *d_z_* = 0.32. For distractor-present trials in Experiment 2, error percentages increased with increasing distance from the high-probability distractor location (5.9%, 8.0%, 9.1%, 10%, 9.4% ms for distances 0 - 4, respectively), *F*(4, 152) = 6.16, *p* < .01, η_p_^2^ = .139, but this effect was no longer significant when the high-frequency position was excluded, *F*(3, 114) = 1.42, *p* = .24, η_p_^2^ = .036.

**Supplemental Material B**

We analyzed judgments of the most frequent distractor position in Experiment 2. The probability of correctly guessing one of eight positions by chance is 12.5%. Out of 39 participants, 17 or 43.6% indicated the correct position, which is above chance performance by binomial test, *p* < .01. This result is comparable to Wang and Theeuwes (2018) who reported 47% correct judgments with a sample size of 32 participants. As in Wang and Theeuwes (2018), the main results were unchanged when participants who had correctly indicated the high-frequency position were excluded.

**References**

Wang, B., & Theeuwes, J. (2018). Statistical regularities modulate attentional capture. *Journal of Experimental Psychology: Human Perception and Performance, 44*(1), 13-17. https://doi.org/10.1037/xhp0000472
